# Femtosecond Laser Direct Writing of Optical Overpass

**DOI:** 10.3390/mi13071158

**Published:** 2022-07-21

**Authors:** Xiaochuan Ding, Yao Zhao, Ali Hassan, Yunlu Sun, Zhishan Hou, Wei Xue, Yu Cao

**Affiliations:** 1 International Science and Technology Cooperation Base for Laser Processing Robot, College of Mechanical and Electrical Engineering, Wenzhou University, Wenzhou 325035, China; 21451439005@stu.wzu.edu.cn (X.D.); alirao@wzu.edu.cn (A.H.); xuewei@wzu.edu.cn (W.X.); 2 State Key Laboratory of Precision Measurement Technology and Instruments, Department of Precision Instrument, Tsinghua University, Beijing 100084, China; zhao-y20@mails.tsinghua.edu.cn (Y.Z.); ylsun@fudan.edu.cn (Y.S.); 3 Oujiang Laboratory (Zhejiang Lab for Regenerative Medicine, Vision and Brain Health), Wenzhou University, Wenzhou 325000, China

**Keywords:** femtosecond laser direct writing, polymer, photonic jumper wire, optical overpass

## Abstract

With the rapid increase in information density, problems such as signal crosstalk and crossover restrict the further expansion of chip integration levels and packaging density. Based on this, a novel waveguide structure—photonic jumper wire—is proposed here to break through the technical restrictions in waveguide crossing and parallel line wrapping, which hinder the integration of photonic chips. Furthermore, we fabricated the optical overpass to realize a more complex on-chip optical cross-connection. Our method and structure promote a series of practical schemes for improving optical chip integration.

## 1. Introduction

The development of microelectronic devices has gone through the research process starting from discrete devices [1] (such as resistors, capacitors, inductors, transistors, etc.) and developed into integrated devices and even relatively large-scale integrated circuits [2]. The development of optical devices (lasers [3,4,5], amplifiers [6], modulators [7], filters [8], waveguide circuits [9], switches, detectors, etc.) will go through a similar process. Photonic integration will be the development trend of photonic technology. In a general sense, photonic integration refers to integrating various active and passive components on the same substrate and the interconnection or communication between them through optical waveguides to form an on-chip optical system with specific functions. Compared with discrete devices, photonic integration can effectively improve the performance, versatility, and reduce the manufacturing cost of existing optical devices, such as a significant reduction in the cost of packaging and beam alignment of discrete devices. In addition, it can significantly improve the stability and reliability of the optical system [10,11]. However, with the increase of integration density and complexity requirements, technical bottlenecks will inevitably be like those of integrated circuits, such as hundreds or even thousands of waveguide crossings (WGX), non-planar circuit topologies [12,13], and the number of WGX often rapidly increasing with the increase in complexity. Therefore, a related photonic device that acts an electronic bonding jumper is required to realize optical interconnections on the chip.

The mainstream micro/nano processing technologies include optical exposure technology [14], ion beam [15,16], electron beam lithography [17,18], nano-imprint technology [19], etching technology, 3D extrusion printing [20], electrochemical machining [21], femtosecond laser direct writing technology (FsLDW) [22,23,24,25] and so on. Among them, FsLDW and 3D printing technology can perfectly adapt to the preparation of photonic jumper wire because of their flexible customizability. However, 3D printing technology has higher requirements of materials, and the existing machining accuracy struggles to meet the high-precision manufacturing requirements of jumper wires. On the other hand, the two-photon femtosecond laser direct writing technology with high-energy pulses directly acts on the interior of the material to realize three-dimensional, nano-scale resolution and maskless processing [26] with arbitrary structure design. Because of nonlinear absorption characteristics [27,28,29], femtosecond laser-induced two-photon direct writing can not only achieve a resolution far beyond the optical diffraction limit [30,31,32] (below 10 nm), but also has a wide range of material processing capabilities, from soft polymer materials to hard materials such as metals, semiconductors, and dielectric materials. Yin et al. presented a large-area periodic nanoripple-structured stainless-steel mesh prepared through a one-step femtosecond laser direct writing process. The as-prepared mesh shows excellent properties of super hydrophilicity and underwater superoleophobicity with low oil adhesion [33]. K.M. et al. discussed the effect of various parameters, such as laser fluence, number of pulses, laser beam polarization, wavelength, incident angle, scan velocity, number of scans, and environment, on the formation of different structures. Furthermore, a guideline for surface structure optimization is provided [34,35].

On-chip interconnection is considered one of the most challenging areas in ultra-large-scale integration. In integrated circuits, to solve the Joule heating and low current-carrying capacity brought by ultra-small sizes, the research focus is mainly on the resistivity and reliability of device materials [36]. The photonic on-chip interconnection technology has no effect on possible loss and current-carrying problems. The current technical difficulty of purely optical integrated chips is still relatively high. The on-chip or off-chip interconnections in optoelectronic integrated chips can be used in ultra-small sizes. A high-precision and controllable free-form optical interconnect structure and common thread-level parallelism in integrated circuits place high demands on on-chip interconnections [37].

Based on this, we propose the fabrication of a photonic jumper wire using two-photon FsLDW to solve the problem of low integration of optical interconnection by point-to-point connection in the three-dimensional space. The commercial photoresist SU-8 was used as the primary material. The span length and height limitations of the jumper after direct polymerization are discussed, and the transmission loss of the prepared jumper wire has been measured. It has been proved that the optical signal can be transmitted by the laser direct writing of jumper wire. Furthermore, we propose an optical overpass structure to realize the more complex on-chip optical cross interconnection, which is an essential step in solving waveguide crossing and parallel line-wrapping. We believe that our proposed method and structure can inspire a series of practical schemes to improve the integration of optical chips.

## 2. Device Design and Fabrication

**Figure 1:**
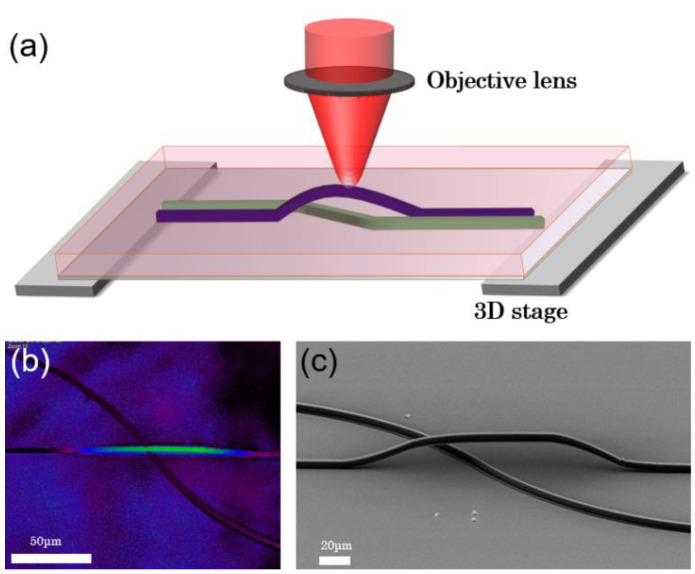
(**a**) The laser processing of photonic jumper wire. (**b**,**c**) Confocal micrograph SEM image of the jumper wire.

The laser processing of photonic jumper wire is shown in Figure 1. SU-8 negative photoresist was the primary material of the waveguide, and cover glass was the substrate. Firstly, the monomer SU-8 (Commercial SU-8 negative photoresist, MicroChem, Japan, Tokyo) was mixed and dissolved in cyclopentanone with a mass ratio of 1:2 to reduce the viscosity of the photoresist, making the photoresist easy to spin-coat, which significantly improved the coating effect and quality. Secondly, the mixed solution was then spin-coated (SmartCoater spin coating instrument, Ansace (China) Co., Ltd.) on the substrate (priorly washed with acetone, and ethanol (volume fraction 75%), the film was prepared by spin-coating at 2000 rpm for 2 min followed, and deionized water (18.2 M Ω cm, 25 °C; water purification system purchased from Milibo)). Finally, the spin-coated sample was baked on a heating stage at 95 °C for 15 min to produce a thicker layer of about 40 microns. In the aforementioned process, cyclopentanone (the organic solvent) volatilized and the remaining photoresist was uniformly distributed over cover glass.

Following laser scanning (with pulse parameters; central wavelength of 800 nm, pulse width of 120 fs, repetition rate of 80 MHz, and power density of 10 mW), the sample was placed on a heating stage (15 min, 95–100 °C), and the acid produced by laser scanning acted as a catalyst to promote photoresist cross-linking to form a dense cross-linking network, which was insoluble in the developer. The unexposed area was not cross-linked and was washed away so the exposed area presents a processing pattern after development. After the sample was cooled to room temperature, the sample was then immersed in an acetone solution (30 s) to develop and remove the uncross-linked area. At last, the sample was rinsed with ethanol and deionized water to obtain the polymer-based photonic jumper wire. It can be seen from the confocal microscope in Figure 1b that the height difference between the two lines formed a three-dimensional structure, which can also be seen from the SEM image. These results preliminarily confirm the feasibility of the waveguide jumper.

## 3. Results and Discussion

The photoresist forms a cross-linking network after laser exposure and baking. The polymer network is flexible during development, so it inevitably collapses if the span length of the jumper wire is too long. Therefore, it is necessary to clarify the length span limit of jumper wire.

**Figure 2:**
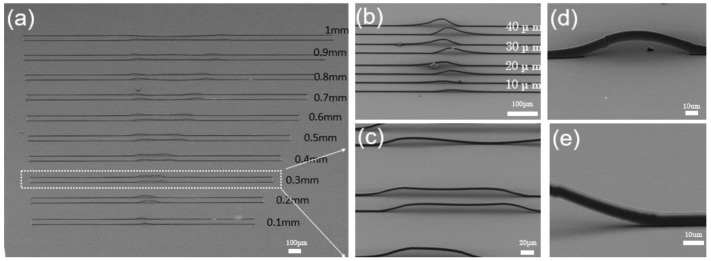
(**a**) SEM images of jumper wires at different lengths. (**b**) Jumper wires with 10–40 μm in height. (**c**) Jumper wires with 0.3 mm in length. (**d**) Jumper images with higher multiples. (**e**) Partial magnification of the jumper.

Figure 2b shows the jumper wires spanning the height from 10 μm to 40 μm. From the initial slight bulge of 10 μm to the structure of 40 μm, similar to the peak, it can be seen that laser direct writing could realize the expected jumper wire fabrication well. Then, the span length limit of the jumper wires is discussed. Figure 2a shows waveguides with spanning lengths from 0.1 mm to 1 mm fabricated separately. Due to the scouring of the development process, as shown in SEM images, and the swelling effect of the photoresist, the structure across the height of 20 μm becomes remarkably steep when the span length is 0.1 and 0.2 mm, which undoubtedly increases the transmission losses of the jumper wire. When the span length is longer, the shape of the jumper wire begins to flatten. When the length is greater than 0.4 mm, the bridge deck structure of the jumper struggles to come out, as the middle begins to collapse, indicating that the supporting force of the jumper structure is struggling to support the gravity of the design center. Therefore, it has been proved that the best span length of jumper wire is about 0.3 mm. We noted that the selected photoresist material determines the current limit length and its polymerization density, which affect the mechanical strength of the polymerized waveguide, so that the length limit can be optimized and selected accordingly. We evaluated the attachment of air dust and faults induced by inappropriate positioning after the manufacturing was completed for the severe scattering generated by the jumper propagation depicted in the Figure 2.

**Figure 3:**
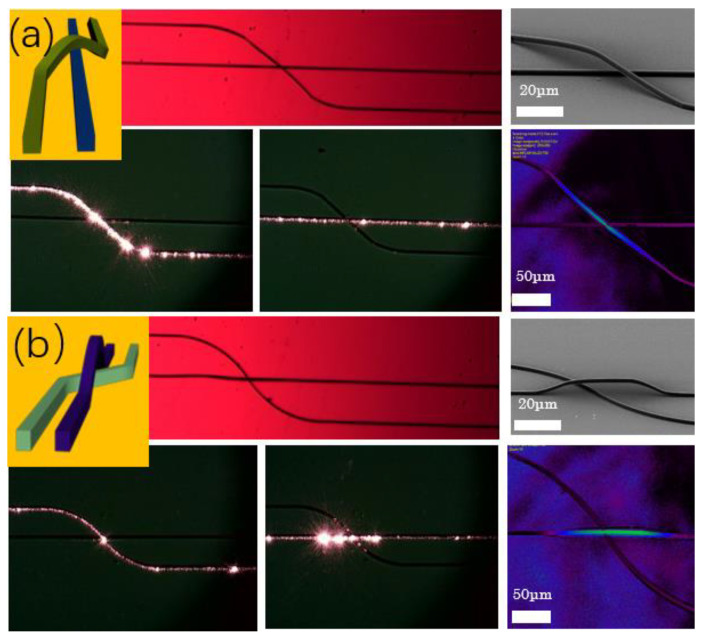
Schematic diagrams and characterization images of (**a**) jumper wire across the straight-line waveguide and (**b**) jumper wire across the curved waveguide.

After the span length was optimized, two kinds of waveguide jumpers with different shapes were designed to solve the complex problems in the integrated optical chip. Since the optical waveguide circuit is like the electronic circuit, most of the paths are parallel straight lines, and we designed two modes of jumper wire. To solve the problem of optical path crossover and integrated switching network in multi-channel parallel processing, we prepared two kinds of photonic jumper wires. Figure 3a shows the schematic diagram and microscope photo of the jumper wire across a straight line. Figure 3b shows the schematic diagram and characterization diagram of a curved jumper wire. Choosing the optimal jumper length means both jumper wires have suitable topography without collapse. Both have good optical performance when coupling light into wires, which shows that the prepared phonic jumper wire has low selectivity and high adaptability in practical application. Then, the transmission loss of the jumper is measured. After testing, we found that the transmission loss of a 1 cm long waveguide with jumper is 10.2 dB/cm, and the transmission loss of a 1 cm long waveguide without jumper is 6.3 dB/cm Therefore, it can be judged that the basic insertion transmission loss of the waveguide is 6.3 dB/cm. Welm M. Patzold et al. demonstrated straight and s-curve waveguides in polymers fabricated by femtosecond laser writing. Several parallel tracks are printed inside the bulk material with a well-defined gap in the middle that forms the waveguide core, with very low propagation losses of 0.3 dB/cm and with no significant bend losses for curve radii of R ≥ 20 mm [31]. The transmission loss of a jumper wire is over one order of magnitude more than that of a typical direct writing waveguide (0.3 dB/cm) generated by laser direct writing. We intend to investigate this more in the future and lower it to a level comparable to that of standard direct writing waveguides. We preliminarily estimated the height in the confocal height scan of the waveguide jumper in Figure 3, which is about 10 μm, we further determined this height through the SEM characterization images in Figure 2 and Figure 3. Therefore, we did not perform subsequent experimental measurements.

**Figure 4:**
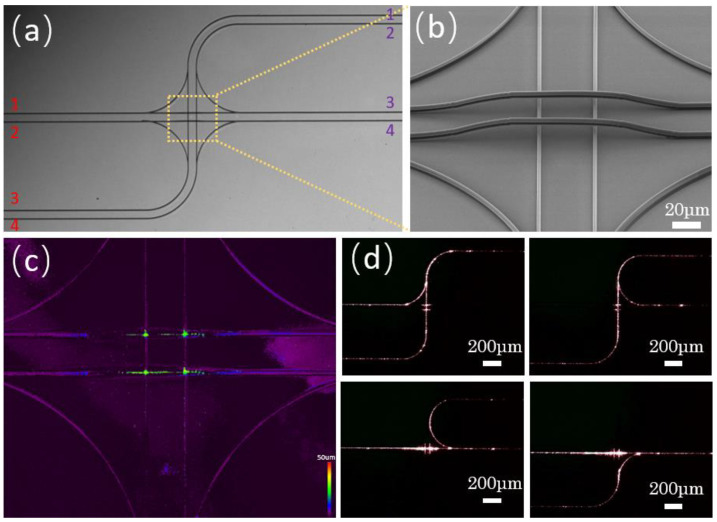
SEM images of (**a**) optical overpass and (**b**) magnified detail. (**c**) Confocal image of the optical overpass. (**d**) Top view of dark field light test of the optical overpass.

After the series of tests above, the data fully support the structural characterization and performance of photonic jumper wires. An optical overpass structure was designed and prepared for further complex applications, as shown in Figure 4. The purple 1–4 represent the input light paths, and the red markers represent the output light paths. Figure 4a–c shows the SEM images and confocal images of the optical overpass. Figure 4d shows the light transmission in the overpass under different incident paths. Such results are in good agreement with the design expectations, and the eight-way optical cross-connection is realized in a small space. The feasibility of an optical overpass in optical interconnection was proved, and it can continue to increase in complexity and integration.

## 4. Conclusions

At present, optical chip interconnection technology is the key to solving the bottleneck of integrated optical chips. Simultaneously, the application potential of FsLDW in integrated optical chip interconnection is being underestimated. We successfully demonstrated the fabrication of three-dimensional photonic jumper wires and the complex integrated optical overpass structure using FsLDW. The structural feasibility and optical performance of the proposed design were well characterized through the detection of the SEM and confocal images. Furthermore, by comparing the transmission loss of the jumper wire and structure normal optical waveguide, it has been concluded that the transmission performance is almost the same as that of the normal optical waveguide, which indicates that photonic jumper wires can be perfectly integrated into existing waveguide structure. On the other hand, the optical overpass design achieved an excellent solution to the space restriction. The current design has great potential as it provides support for solving the waveguide intersection in the future integrated photonic chip for perfecting the actual three-dimensional complex waveguide array inside the photonic chip.
